# Visual, visuomotor, and kinesthetic abilities in football players: their relationship with sport-specific performance and trainability

**DOI:** 10.3389/fnins.2026.1930426

**Published:** 2026-08-24

**Authors:** Congcong Weng, Dongxu Gao, Xiushen Dong, Yuqing Gao, Qiangquan Wang, Jiayu Wang, Junzhe Zou, Lihao Shan, Honglei Zhang

**Affiliations:** 1 Department of Physical Education and Military Training, Jiaxing University, Jiaxing, China; 2 Division of Sports Science and Physical Education, Tsinghua University, Beijing, China; 3 School of Physical Education, Hunan Normal University, Changsha, China; 4 School of Physical Education, Wuhan Sports University, Wuhan, China; 5 China Institute of Sport and Health, Ningxia Normal University, Guyuan, China; 6 School of Physical Education, Hubei University, Wuhan, China; 7 School of Physical Education, Yanbian University, Yanji, China; 8 School of Physical Education, Shanghai University of Sport, Shanghai, China; 9 Pinghu Normal College, Jiaxing University, Pinghu, China

**Keywords:** athletic performance, perceptual-cognitive training, proprioception, soccer, sports vision

## Abstract

Success in football (soccer) rests on physical and technical capacity and on the perceptual and sensorimotor systems that let players read the game, act quickly, and control the body and the ball. This review examines three linked ability domains: visual (the quality and use of visual input, from static and dynamic acuity to peripheral awareness, object tracking, and visual search), visuomotor (the coupling of perception to action, including reaction time, eye-foot coordination, and visually guided movement), and kinesthetic (proprioception, joint position sense, kinesthetic differentiation, and balance). For each domain we ask how it is measured, how it relates to match-relevant performance, and whether it can be trained. Football players generally outperform non-athletes, and several markers separate players by level, position, and age. Trainability is consistent for targeted laboratory measures across stroboscopic, light-based, augmented-feedback, perceptual-cognitive, and proprioceptive-coordinative programs, but transfer to on-field performance is weaker and less certain, and laboratory tests frequently fail to predict in-game behavior. We argue that the three domains form a single perception-action continuum rather than separate capacities, that assessment and training should represent real match demands, and that sex, limb dominance, maturation, fatigue, and injury status act as moderators. Adequately powered, field-validated trials remain the decisive next step. Practical implications for talent identification, monitoring, and training design are set out. The evidence base is drawn overwhelmingly from male players, so these conclusions apply to men’s football unless otherwise stated.

## 1 Introduction

Performance in football, the association-football code commonly called soccer, is multifactorial. Physical, technical, tactical, and psychological qualities each contribute, and no single attribute explains success at the highest level (Vestberg et al., 2017). Beneath the technical and tactical surface sits a perceptual and sensorimotor substrate: players must continuously sample a moving scene, extract the information that matters, and convert it into fast, accurate action. Football is an externally paced invasion sport played in an open, changing environment, so the demand is not only to move well but to perceive early and choose under time pressure (Gredin et al., 2018). Skilled players meet this demand by combining basic visual capacity with the cognitive machinery that interprets what is seen and the sensorimotor control that turns intention into movement.

A long-standing distinction separates visual “hardware,” the optical and oculomotor functions that set image quality, from visual “software,” the perceptual-cognitive skill that interprets the image (DeCouto et al., 2024). This review places three constructs on a single perception-to-action pathway. Visual ability is the input stage, spanning static and dynamic acuity, contrast sensitivity, peripheral perception, eye movements, multiple-object tracking (MOT), and visual search or anticipation. Visuomotor ability is the coupling stage, linking perception to executed movement through reaction time (RT), eye-hand and eye-foot coordination, and visually guided action. Kinesthetic ability is the feedback stage that closes the loop, comprising proprioception, joint position sense, force sense, kinesthetic differentiation, and dynamic balance. Detailed definitions are given within each section.

These domains have grown up in largely separate literatures: optometry and sports vision, motor control, sports medicine and rehabilitation, and sport psychology. That separation has left two practically important questions poorly resolved. First, how strongly does each domain relate to actual football performance rather than to laboratory tasks? Normative visual data can rank professional players, yet laboratory perceptual-cognitive scores often predict in-game behavior weakly (Jorge et al., 2026; Van Maarseveen et al., 2018). Second, can these abilities be trained in a way that reaches the pitch? Intervention work is expanding fast, but the balance of near-transfer to trained tasks against far-transfer to competition is contested (Zhu et al., 2024). Research on match decision-making alone has accumulated rapidly over the past 15 years, which makes an integrated reading useful rather than premature (Paucar Uribe et al., 2025). For practitioners, the same uncertainty carries direct costs: perceptual and sensorimotor measures are already used for talent identification, readiness monitoring, injury prevention, and return-to-play decisions, yet advice drawn from one specialty rarely accounts for what the others have shown.

**FIGURE 1:**
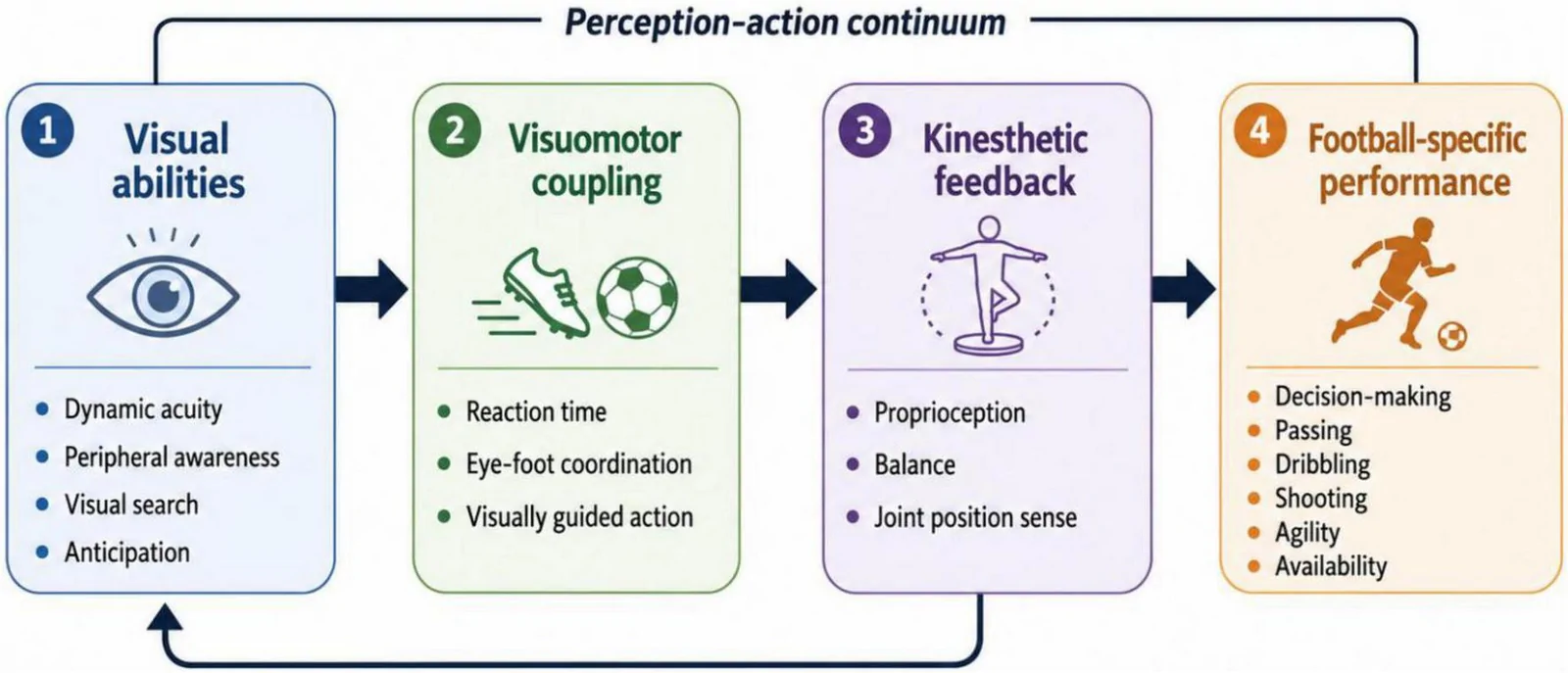
Summarizes the proposed perception-action continuum through which visual, visuomotor, and kinesthetic abilities contribute to football-specific performance.

We address two guiding questions for each domain, its relationship with sport-specific performance and its trainability, and we organize the paper around them: every domain is examined for assessment, performance link, and trainability, followed by an integrative synthesis and a critical appraisal of methods and evidence. Scope covers association football across youth-to-senior ages and competitive levels and includes both outfield players and goalkeepers, with sex, age, and level noted where the evidence allows. What this review adds beyond existing single-domain syntheses is a unifying position: the three domains are not separable capacities but coupled stages of one perception-action system, and that framing explains why laboratory gains and match performance so often diverge. It also clarifies what a practitioner can and cannot infer from a single perceptual-motor test score, which is the point at which this science meets applied decisions about selection, monitoring, and rehabilitation. The conceptual framework of this review is summarized in Figure 1, which presents visual, visuomotor, and kinesthetic abilities as linked stages of a single perception-action continuum leading to football-specific performance.

### 1.1 Scope and approach to the literature

This paper is a critical narrative review rather than a systematic review. It was not registered and does not follow PRISMA. Our aim was to cover three domains that are usually reviewed separately, and a systematic protocol built around a single answerable question would not have allowed that breadth. The cost of this choice is that the synthesis presented below is structured but not exhaustive. Searches were run in PubMed, Web of Science, Scopus, and SPORTDiscus, covering each database from its inception up to July 2026. Reference lists of the included papers and of recent reviews were checked by hand, and forward citation tracking was used to locate later work. The search combined three blocks of terms with AND, with the terms inside each block combined with OR. The population block was football OR soccer OR footballer OR goalkeeper. The domain block was vision OR visual acuity OR dynamic visual acuity OR contrast sensitivity OR peripheral perception OR multiple object tracking OR visual search OR anticipation OR gaze OR perceptual-cognitive OR reaction time OR visuomotor OR eye-foot coordination OR sensorimotor OR proprioception OR joint position sense OR kinesthetic OR kinaesthetic OR balance OR postural control. The outcome block was performance OR expertise OR skill OR talent OR training OR intervention OR transfer OR injury.

Studies were included if they were published in a peer-reviewed journal in English, tested human participants, reported at least one measure of visual, visuomotor, or kinesthetic ability, and either related that measure to football performance, expertise, or player availability, or tested the effect of a training intervention on it. Original studies, systematic reviews, and meta-analyses were all eligible. Conference abstracts, unpublished theses, and opinion pieces without data were excluded. Evidence from populations other than footballers was used only where it establishes a mechanism for which football-specific data are not yet available, as in the chronic ankle instability work on sensory reweighting, and these cases are identified as non-football in the text and in the tables.

Tables 2, 3 list the retrieved records meeting these criteria that reported, respectively, a quantified association between a domain measure and a football performance, expertise, or availability outcome, and a pre-post or controlled effect of a training intervention on a domain measure. Where several reports drew on a single cohort, the most complete was retained. Null, negative, and internally inconsistent results were eligible on the same terms as positive ones and are tabulated alongside them. Because screening was not duplicated and no protocol was registered, the tables are a structured map of the evidence base rather than an exhaustive inventory, and the evaluative statements drawn from them in Sections 2 to 5 are framed accordingly.

No formal risk-of-bias instrument was applied to the included studies. The appraisal presented in section “6 Methodological considerations and limitations” is therefore a narrative judgment based on stated criteria of design, sampling, measurement quality, and choice of comparison group. It should be read on those terms rather than as a scored assessment.

## 2 Visual abilities in football

### 2.1 Assessment and characteristic visual profiles

Sport-vision assessment follows the hardware-software split. Hardware measures index the quality of the retinal image and the ocular systems that stabilize it: static acuity, dynamic visual acuity (DVA), contrast sensitivity, stereopsis, and accommodative and vergence function. DVA is the more football-relevant of the acuity measures because the sport is built around a moving ball and moving players, and DVA is itself sensitive to target trajectory and contrast rather than being a single fixed value (Quevedo-Junyent and Argilés, 2024). Software measures index how the image is used: peripheral perception, MOT including three-dimensional variants (3D-MOT), visual search and gaze control, anticipation and advance-cue use, and pattern recall. The specific instruments used for each construct, with their typical metrics, are consolidated in Table 1, so the emphasis here is on categories and rationale rather than a catalogue of devices.

**TABLE 1:** Assessment of visual, visuomotor, and kinesthetic abilities in football: constructs, representative tests, and typical metrics.

Domain and sub-construct	Representative test or paradigm (typical metric)	Example instrument	References
Visual: static and dynamic acuity	Optotype chart; moving-optotype dynamic acuity (logMAR; threshold velocity)	Snellen or logMAR chart; dynamic visual acuity test	Jorge et al., 2026; Quevedo-Junyent and Argilés, 2024
Visual: contrast sensitivity and stereopsis	Grating or letter contrast; stereo test (log units; arcsec)	Contrast chart; distance stereoacuity test	Jorge et al., 2026
Visual: peripheral perception	Peripheral stimulus detection (span; reaction time)	Standardized computer battery; light-based vision boards	Rodrigues et al., 2025; Zwierko M. et al., 2020
Visual: multiple-object tracking	Two- or three-dimensional tracking speed threshold; learning rate	NeuroTracker (3D-MOT)	Chermann et al., 2018; Lysenko-Martin et al., 2020
Visual: visual search and anticipation	Eye tracking with temporal or spatial occlusion (fixation number and duration; prediction accuracy)	Mobile eye tracker; film or virtual-reality display	Gredin et al., 2018; Zwierko T. et al., 2019
Visuomotor: reaction time (simple and choice)	Stimulus-response latency (ms)	Standardized computer battery	Neto et al., 2022; Schumacher et al., 2018
Visuomotor: eye-hand and eye-foot coordination	Instrumented target passing or kicking (accuracy, timing)	COGNIFOOT instrumented passing system	Hicheur et al., 2020
Visuomotor: visually guided control and adaptation	Postural and visuomotor tracking or rotation (spatiotemporal error)	Dual force platform with visual feedback	Vouras et al., 2023
Visuomotor: bilateral force control	Isometric force matching (accuracy, variability, symmetry)	Force or isokinetic dynamometry	Lee T. L. et al., 2024
Kinesthetic: joint position sense	Active or passive angle reproduction at knee or ankle (absolute and relative error)	Video-marker analysis; dynamometer	Azevedo et al., 2025
Kinesthetic: kinesthetic differentiation	Graded ball strike to a target (spatial error)	Ball-landing target sectors	Boraczyński et al., 2019
Kinesthetic: dynamic balance and postural control	Reach-based and single-leg tests; center-of-pressure (reach distance; stability index)	Y-Balance Test or Star Excursion Balance Test; force platform; Kinesthetic ability trainer	Aytar et al., 2012; Snyder and Cinelli, 2020

Two features of the software layer matter for football. Object-tracking capacity, measured as a speed threshold and a learning rate, engages the same attentional processes that are disrupted by concussion, which is why tracking tasks double as clinical monitoring tools (Chermann et al., 2018; Lysenko-Martin et al., 2020). Anticipation, measured with temporal or spatial occlusion and eye tracking, is where expertise is most visible: skilled players extract advance information from an opponent’s kinematics and combine it with prior knowledge of that opponent’s tendencies, an integration that novices perform poorly (Gredin et al., 2018). Even without a sport-specific display, players show more efficient oculomotor behavior than non-athletes, with faster saccades during visual search (Zwierko T. et al., 2019).

Gaze control and pattern recall complete the software layer. Skilled performers deploy a more efficient search, fixating informative regions, and they recall structured game patterns better than novices, a memory advantage that indexes how they encode the scene rather than how well they see it (Gredin et al., 2018). Because these are learned, knowledge-based skills, they are the components most plausibly open to training, and also the ones most likely to be specific to a sport and even to an individual opponent.

The central methodological problem is ecological validity. Generic laboratory tasks measure capacities in isolation, but tracking and search in a real match depend on how players are distributed in space and on sport-specific movement patterns, features that generic stimuli omit. Soccer-specific virtual environments and web-delivered tasks are one response, allowing player movements drawn from real matches to drive the test (Vu et al., 2022). Immersive and peripheral paradigms extend this further by probing how players judge configurations that appear away from the point of fixation (Klatt et al., 2020).

### 2.2 Relationship with sport-specific performance

Expertise differences in the visual domain are real but concentrated in the software layer. Experts anticipate the direction of an opponent’s action earlier and use advance cues that novices ignore, and this advantage survives when prior information conflicts with unfolding kinematics (Gredin et al., 2018). In goalkeeping, high-skill performers time their action to reliable late information such as the placement of the kicker’s non-kicking leg, indicating that perception is tuned to the specific information a task affords (Zheng R. et al., 2021). Hardware capacity does discriminate at the elite end, where normative acuity, binocular, and visuo-cognitive thresholds separate professionals into performance tiers, but this ranking is descriptive rather than predictive of match outcomes (Table 2).

**TABLE 2:** Studies relating visual, visuomotor, and kinesthetic abilities to football-specific performance and status.

Domain(s)	Sample and key ability measure	Main performance-related finding	References
Visual	262 professional players; acuity, binocular, and visuo-cognitive battery	Normative thresholds separated professionals into five performance tiers	Jorge et al., 2026
Visual and perceptual-cognitive	Skilled players; anticipation, decision-making, pattern recall with gaze	Laboratory scores did not predict in-situ on-field performance	Van Maarseveen et al., 2018
Visual	Expert vs novice players; contextual priors plus opponent kinematics, gaze	Experts fused priors with kinematics; priors misled novices, not experts, on incongruent trials	Gredin et al., 2018
Visual and cognitive	Young elite players; core and higher executive function	Executive-function scores correlated with goals scored across a season	Vestberg et al., 2017
Visual and cognitive	Elite players; visual skills and executive function	Near-far quickness related most strongly to selective attention and flexibility	Knöllner et al., 2022
Visual and cognitive	Elite youth; attention window, tracking, working memory	Basic cognitive functions correlated with sport-specific motor skills	Scharfen and Memmert, 2019
Cognitive and motor	Elite vs low-division youth; inhibition and agility	Higher-division players showed better inhibitory control and soccer agility	Lovecchio et al., 2021
Visuomotor	Talented players by age and position; reaction time and attention	Reaction and attention profiles differed by position and age	Schumacher et al., 2018
Visuomotor and kinesthetic	Players vs non-athletes; single-leg balance with choice reaction	Players kept superior dynamic single-leg stability under decision load	Snyder and Cinelli, 2020
Visuomotor	Players vs controls; bilateral lower-limb force control	Players showed more symmetric and flexible interlimb force coordination	Lee T. L. et al., 2024
Visuomotor	Goalkeepers vs non-athletes; whole-body visuomotor rotation	Goalkeepers adapted faster to a novel transformation in a stable environment	Vouras et al., 2023
Visual	Players vs non-athletes; saccadic visual search with strenuous effort	Players produced faster saccades; exertion reduced oculomotor efficiency	Zwierko T. et al., 2019
Kinesthetic	Unilateral chronic ankle instability meta-analysis; kinesthesia and balance	Uninjured limb also showed kinesthesia and balance deficits	Hu et al., 2024
Visuomotor	Collegiate players; light-based reaction vs field reactive agility	Light-based reaction speed did not predict field reactive agility	Broodryk et al., 2025

Studies were identified by the search described in section “1.1 Scope and approach to the literature” and are those meeting the inclusion criteria that reported a quantified association between a visual, visuomotor, or kinesthetic measure and a football performance, expertise, or availability outcome. Null and negative findings were eligible on the same terms as positive findings. The table maps the evidence base and is not an exhaustive inventory.

The clearer performance signal comes from visual-cognitive integration. Executive function relates to competitive output, including goals scored across a season, and specific visual skills such as near-far quickness map onto selective attention and cognitive flexibility rather than onto general intelligence (Knöllner et al., 2022; Vestberg et al., 2017). Basic cognitive functions also track sport-specific motor skill in elite youth, and combined cognitive and agility screening separates higher-division from lower-division players (Lovecchio et al., 2021; Scharfen and Memmert, 2019). These relationships are moderated by development and role: position and age shape reaction and attention profiles, and the contribution of perceptual-cognitive skill to reactive performance strengthens through mid-adolescence (Horníková et al., 2025; Schumacher et al., 2018).

Two cautions apply. The designs are overwhelmingly correlational, so direction of causation is unproven, and laboratory perceptual-cognitive scores predict in-situ, on-field performance weakly and inconsistently (Van Maarseveen et al., 2018). Weighing these strands, the associations assembled in Table 2 that are strongest and most consistently replicated are cognitive rather than optical: executive function and attention-linked visual skills relate to competitive output more consistently than acuity or stereopsis, yet all of this evidence is correlational, and the one design in this set that tested laboratory perceptual-cognitive scores against actual in-game behavior found little predictive value (Van Maarseveen et al., 2018; Vestberg et al., 2017). The study-level samples, measures, and directions of effect behind these statements are given in Table 2.

### 2.3 Trainability

Targeted visual and perceptual-cognitive capacities respond to training. Stroboscopic protocols, which intermittently occlude vision, improve anticipation beyond control conditions (Fortes et al., 2025), and even a single session can sharpen anticipatory timing (Smith and Mitroff, 2012). Perceptual training of ball-direction prediction improves goalkeepers and novices alike, whether the relevant cues are highlighted explicitly or left implicit (Faquin et al., 2024). Training gaze itself can help as well: a quiet-eye program improved penalty-kick accuracy against a placebo, though the advantage did not survive the added anxiety of a shoot-out, which shows how fragile perceptual gains can be under competitive pressure (Wood and Wilson, 2011). Manipulating the visual environment through light-based control of processing speed improves DVA, reaction, and peripheral accuracy in professionals (Rodrigues et al., 2025), on-field perceptual-cognitive drills improve peripheral reaction (Schumacher et al., 2020), pattern-recall training transfers to retention of tactical patterns (Schorer et al., 2018), and autonomic biofeedback improves visual selective attention (Rusciano et al., 2017). A pattern separates these interventions: those delivered inside a football task or with a sport-specific response tend to move on-field measures, whereas purely off-field visual drills reliably change the trained capacity but have rarely been tested against match performance (Rodrigues et al., 2025; Schumacher et al., 2020). Intervention designs, doses, and effects are set out in Table 3. Near-transfer to the trained task is robust across these studies; whether the gains reach match performance is the unresolved question developed in section “5 Integration, transfer, and the perception-action continuum.”

**TABLE 3:** Intervention studies on the trainability of visual, visuomotor, and kinesthetic abilities in football and related athletes.

Population and intervention (dose)	Effect on targeted ability	Transfer to performance or availability	References
Professional soccer; light-based (Okkulo) visual-processing training, 6 wk	Improved dynamic visual acuity, recognition, sensory and motor reaction, peripheral accuracy	On-field transfer not yet tested	Rodrigues et al., 2025
Soccer players; stroboscopic vision training, 8 wk	Anticipation improved beyond control; tracking and decision-making rose in both groups	Field transfer untested	Fortes et al., 2025
General participants; stroboscopic training, single session	Anticipatory timing improved and was briefly retained	Laboratory task; transfer not assessed	Smith and Mitroff, 2012
U14-U15 elite; augmented visuo-auditory feedback passing, 17 d	Passing accuracy, reactiveness, and global performance improved (about +22%)	Gains on a soccer-specific passing task	Hicheur et al., 2020
Youth elite; on-field perceptual-cognitive training, 8 wk	Peripheral reaction time improved, driven by right-footed players	Sport-specific on-field measure improved	Schumacher et al., 2020
Amateur players; visuomotor training with or without tDCS, 5 sessions	Choice reaction time reduced in kicking and reaching limbs	Partial transfer to the non-trained limb	Neto et al., 2022
Junior players; pattern-recall augmented training	Pattern recall improved at retention	Field tactical transfer not directly shown	Schorer et al., 2018
Prepubertal boys; 12-month proprioceptive-coordinative program	Balance, kinesthetic differentiation, and other motor skills improved	Improved soccer-specific motor tasks	Boraczyński et al., 2019
Amateur men; FIFA 11+ vs proprioceptive program, 6 wk	Knee joint position sense and countermovement jump favored the proprioceptive program	Dynamic balance improved in both	Navarro-Santana et al., 2020
Male youth; FIFA 11+, 10 wk	Dynamic knee valgus reduced; dynamic balance unchanged	Biomechanical risk reduced; limited motor transfer	Soussi et al., 2025
Preadolescent players; speed-agility-quickness vs small-sided games, 4 wk	Inhibition, perceptual speed, and sprint improved	Comparable across programs	Trecroci et al., 2022
Adult amateur; motor-cognitive agility with object tracking, 6 wk	Agility and cognitively loaded dribbling improved	Transferred to football-specific tests; no added benefit of tracking over agility	Friebe et al., 2024
Chronic ankle instability; balance training with stroboscopic glasses, 4 wk	Postural control improved; visual reliance reweighted	Vision-to-proprioception bridge; clinical gain	Lee H. et al., 2022
Athletes including soccer; neuromuscular and balance ACL-prevention programs	Programmatic, not a single ability	ACL rupture risk roughly halved	Watson et al., 2026

Selection as for Table 2 and as described in section “1.1 Scope and approach to the literature” Trials in which control groups improved comparably, and trials reporting no transfer, are included. Entries drawn from non-football populations are identified in the first column.

On balance, visual hardware differs only modestly between players and non-athletes, whereas software measures such as anticipation and executive-loaded visual skill track expertise more closely, yet neither forecasts match behavior reliably on its own.

## 3 Visuomotor abilities in football

### 3.1 Assessment and characteristic profiles

Visuomotor ability is perception-action coupling, the bridge from visual software to executed movement. The constructs assessed are simple and choice RT, eye-hand and eye-foot coordination, visuomotor tracking, visually guided reaching and interception, anticipatory postural adjustments, and sensorimotor integration under visual load. Football places unusual weight on the eye-foot channel rather than the eye-hand channel that dominates most laboratory work, and on limb lateralization, because the two feet are rarely equivalent in a task such as passing or striking. The instruments used for each construct are listed in Table 1.

Assessment paradigms span computer-based batteries and instrumented sport tasks. Standardized batteries quantify latency, peripheral perception, and coordination and reveal position- and age-linked differences in players (Schumacher et al., 2018). Instrumented passing systems capture accuracy and timing when a player must perceive a moving target, anticipate its path, and adjust pass direction and power, giving a football-specific index of cognitive-motor performance (Hicheur et al., 2020). Bilateral force-control tasks isolate how the two lower limbs share and coordinate force output (Lee T. L. et al., 2024). Whole-body visuomotor-rotation and postural-tracking tasks probe how quickly a performer remaps a visual signal onto a motor response, a demand close to reacting to deceptive movement (Vouras et al., 2023). Choice eye-foot reaction can also be measured directly as part of a visuomotor test set (Neto et al., 2022).

Two features make the football case distinctive. The eye-foot channel is slower and less rehearsed than the eye-hand channel that dominates laboratory motor control, so tools designed for hand tasks do not transfer cleanly, and the lower limb must serve locomotion and ball control at the same time. Limb dominance compounds this, because the preferred and non-preferred feet differ in accuracy and in how readily a trained response carries from one limb to the other, so a single-limb test can misrepresent a player’s functional visuomotor capacity (Neto et al., 2022).

### 3.2 Relationship with sport-specific performance

Players show faster and more accurate visuomotor responses and superior interlimb coordination than non-athletes, with more symmetric and more flexible sharing of force between the feet (Lee T. L. et al., 2024). Under a decision load, players maintain better single-leg stability during a reaching task, indicating that eye-foot control and balance are used together rather than separately (Snyder and Cinelli, 2020). Goalkeepers adapt to a novel whole-body visuomotor transformation faster than non-athletes, but only in a stable visual environment, which points to sport-specific priors rather than a general motor advantage (Vouras et al., 2023). The speed of visuomotor, and specifically eye-foot, processing contributes measurably to dynamic postural stability (Zwierko M. et al., 2020). Reliance on this coupling is exposed when it is removed: restricting visual feedback degrades dribbling, and does so most in the fastest dribblers, who depend on continuous visual control of the ball (Fransen et al., 2017). The role of visuomotor integrity in performance and in return-to-play is shown by concussion, which disrupts visually guided motor action (Fueger and Huddleston, 2018). Two inferences follow. The goalkeeper result is bounded, since the adaptation advantage appears in a stable environment but not a variable one, so it reflects learned, context-specific transformations rather than a general capacity to remap vision onto movement (Vouras et al., 2023). The player-versus-non-athlete contrasts, though consistent, rest on comparisons that cannot separate selection from training, so they show that visuomotor coupling distinguishes players without showing that improving it would raise performance. Study-level details appear in Table 2.

### 3.3 Trainability

Visuomotor ability is among the more clearly trainable targets. Augmented visuo-auditory feedback during a passing task improved passing accuracy, reactiveness, and global performance by about a fifth relative to normal training (Hicheur et al., 2020). Visuomotor training reduced choice RT, with an additive effect when paired with transcranial direct current stimulation (tDCS) and with partial carry-over to the non-trained limb (Neto et al., 2022). On-field perceptual-cognitive training improved peripheral reaction, and light-based training improved sensory and motor reaction (Rodrigues et al., 2025; Schumacher et al., 2020). Combined cognitive-motor work also produces gains: a short speed, agility, and quickness (SAQ) program improved cognitive and physical measures comparably to soccer-specific small-sided games, and motor-cognitive agility training transferred to football-specific test performance (Friebe et al., 2024; Trecroci et al., 2022). Two qualifications recur across these trials, dose and specificity of the trained response, and lateralized effects tied to limb dominance. The trials also differ in how close their outcomes sit to the game: augmented feedback and motor-cognitive agility produced gains on soccer-specific tasks, whereas the reaction-time reductions from added brain stimulation are, so far, laboratory effects whose match relevance is untested (Friebe et al., 2024; Hicheur et al., 2020; Neto et al., 2022). The practical reading is that coupling the trained response to a football action, rather than the training modality itself, is what separates transfer from a laboratory gain. The intervention data are given in Table 3.

The strongest visuomotor signal is not raw reaction speed but the quality of eye-foot coupling and interlimb coordination, which is where players most clearly exceed non-athletes and where training gains are most convincing.

## 4 Kinesthetic abilities in football

### 4.1 Assessment and characteristic profiles

Kinesthesis, or proprioception, is the set of senses that report joint position (joint position sense, JPS), movement, and force, together with kinesthetic differentiation, the grading and dosing of movement, and dynamic balance and postural control as their functional expression. Assessment covers active and passive joint-position reproduction at the knee and ankle, thresholds to detection of passive motion, force reproduction, and balance and postural-stability tests using single-leg stance, reach-based protocols, and center-of-pressure recording on force platforms, alongside ball-landing tasks that quantify kinesthetic differentiation. The specific tests and metrics are consolidated in Table 1.

Proprioceptive acuity is not a fixed trait. It is sensitive to fatigue and injury and is modifiable by acute interventions: simulated match fatigue degrades explosive neuromuscular function and the whole-body response to balance disturbances (Behan et al., 2018), and prolonged static stretching can shift knee JPS, an effect that depends on stretch duration (Azevedo et al., 2025). This lability means that a single proprioceptive measurement describes a state as much as a capacity, which has consequences for how the domain should be tested and interpreted. In practice, proprioceptive tests should be timed relative to fatigue and recent activity, because a deficit recorded after exertion may reflect transient neuromuscular decline rather than a stable trait (Behan et al., 2018). Acute preparation choices matter for the same reason, since interventions applied shortly before performance can move joint position sense in either direction depending on their type and dose (Azevedo et al., 2025).

### 4.2 Relationship with sport-specific performance

Players display superior single-leg balance and more effective use of proprioceptive information than non-athletes, which supports dynamic stability during actions such as striking, shielding, and changing direction (Snyder and Cinelli, 2020). Kinesthetic differentiation and balance are the plausible substrate for graded ball actions, first touch, and striking accuracy, although this link is inferred from balance and differentiation measures rather than demonstrated directly against skill outcomes. The most concrete performance consequence of kinesthetic function is availability, which is itself a determinant of team performance. Proprioceptive and neuromuscular deficits are risk factors for the non-contact injuries that remove players from competition, and the deficits are not confined to an injured limb: in unilateral chronic ankle instability (CAI), the uninjured limb also shows impaired kinesthesia and balance (Hu et al., 2024). Interlimb asymmetry in landing mechanics marks elevated anterior cruciate ligament (ACL) risk in female players, a group for whom this evidence is comparatively scarce (Katircioglu et al., 2025), and residual deficits after ankle sprain are strongly tied to inadequate rehabilitation and premature return (Marín Fermín et al., 2023). The evidential asymmetry here is notable: the link between kinesthetic ability and skilled ball actions is largely inferred from balance and differentiation measures, whereas the link to availability is supported by prospective and meta-analytic injury data, so the domain’s best-evidenced contribution to team success runs through staying on the pitch rather than through refined technique (Hu et al., 2024). Study-level findings are given in Table 2.

### 4.3 Trainability

Kinesthetic ability is highly trainable. A 12-month proprioceptive-coordinative program improved balance, kinesthetic differentiation, and other soccer-specific motor skills in prepubertal boys (Boraczyński et al., 2019), and warm-up programs with a proprioceptive component alter knee JPS and dynamic balance in amateur players (Navarro-Santana et al., 2020). The developmental timing of this trainability matters, since the largest and most durable coordinative gains are reported in prepubertal and adolescent players, consistent with a sensitive period for perceptual-motor development, which argues for introducing proprioceptive-coordinative work early rather than treating it as remedial (Boraczyński et al., 2019). Balance and neuromuscular training reduce injury: meta-analytic evidence shows that balance- and proprioception-based programs lower knee-injury risk, and that structured neuromuscular programs roughly halve ACL rupture risk (Watson et al., 2026; Zheng G et al., 2025), and comparable approaches restore function in CAI (Tedeschi et al., 2024). The effect on capacity is not uniform, however. Not every program improves balance: a 10-week FIFA 11+ intervention reduced dynamic knee valgus without changing dynamic balance, whereas a neuromuscular program emphasizing spatial orientation and landing control improved both balance and strength (Hasani Chenari et al., 2025; Soussi et al., 2025). This inconsistency argues that the specific content of a program, not its label, determines which kinesthetic capacities improve.

The domain also provides the clearest bridge between vision and proprioception. Training balance while vision is intermittently occluded reweights sensory reliance and improves proprioceptive postural control in CAI, a mechanism developed in “section 5 Integration, transfer, and the perception-action continuum” (Lee H. et al., 2022). Intervention designs and outcomes are given in Table 3.

**FIGURE 2:**
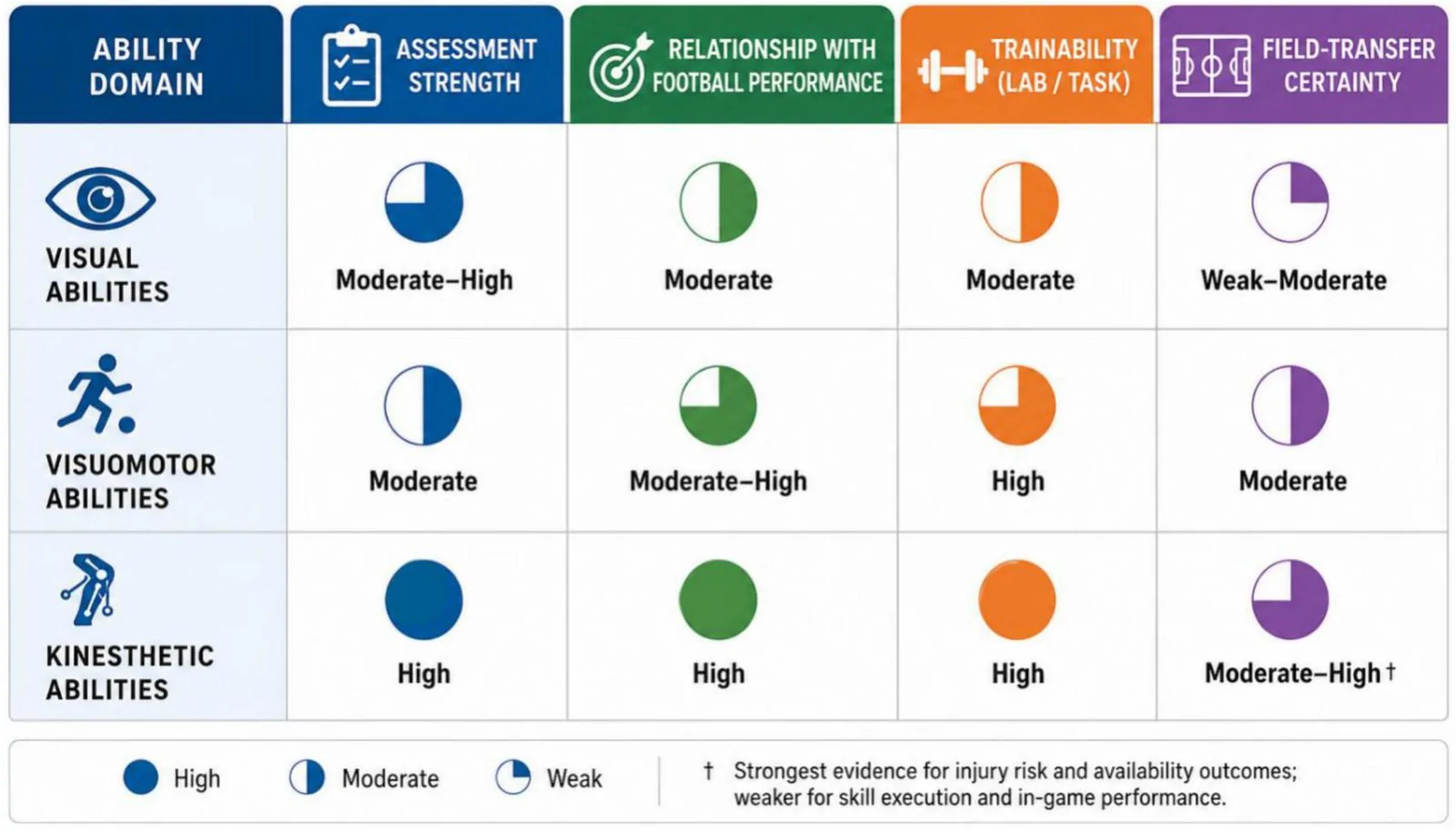
Evidence map of visual, visuomotor, and kinesthetic abilities in football.

Kinesthetic ability is the most trainable of the three domains and the one with the clearest link to a concrete outcome, injury availability, even though its contribution to skilled ball actions is inferred more than it is measured. The comparative strength and limitations of the evidence across visual, visuomotor, and kinesthetic domains are summarized in Figure 2.

## 5 Integration, transfer, and the perception-action continuum

The three domains are interdependent rather than separable. Vision supplies the information that visuomotor processes convert into action, and kinesthetic feedback calibrates that action and updates the next perceptual sample. The clearest empirical sign of this coupling is sensory reweighting between vision and proprioception: when vision is degraded during balance training, performers shift their reliance toward proprioceptive input and improve postural control, and CAI patients who depend more on vision are the ones most affected when that input is disturbed (Han et al., 2022; Lee H. et al., 2022). Vision also contributes to the anticipation of ground-reaction forces during landing, so manipulating it changes movement output even in tasks that look purely motor (Kroll et al., 2023). Consistent with a single control loop, the speed of visuomotor processing feeds directly into postural stability (Zwierko M. et al., 2020). These findings support treating the domains as one perception-action system in which the same information is used to perceive, to act, and to control the body.

Expertise is distributed across this continuum but not uniformly. Experts differ from non-experts in visual search and anticipation, in visuomotor adaptation, and in proprioceptive and balance control (DeCouto et al., 2024; Snyder and Cinelli, 2020; Vouras et al., 2023). The uneven distribution matters for interpretation: superiority on one stage does not guarantee superiority on another, and, more importantly, laboratory superiority does not guarantee field superiority (Van Maarseveen et al., 2018). This unevenness also cautions against composite perceptual-motor scores that average across stages, because a player may track well yet read the game poorly, or anticipate well yet lack the balance to execute, and averaging obscures the individual profile that decides performance (DeCouto et al., 2024).

**FIGURE 3:**
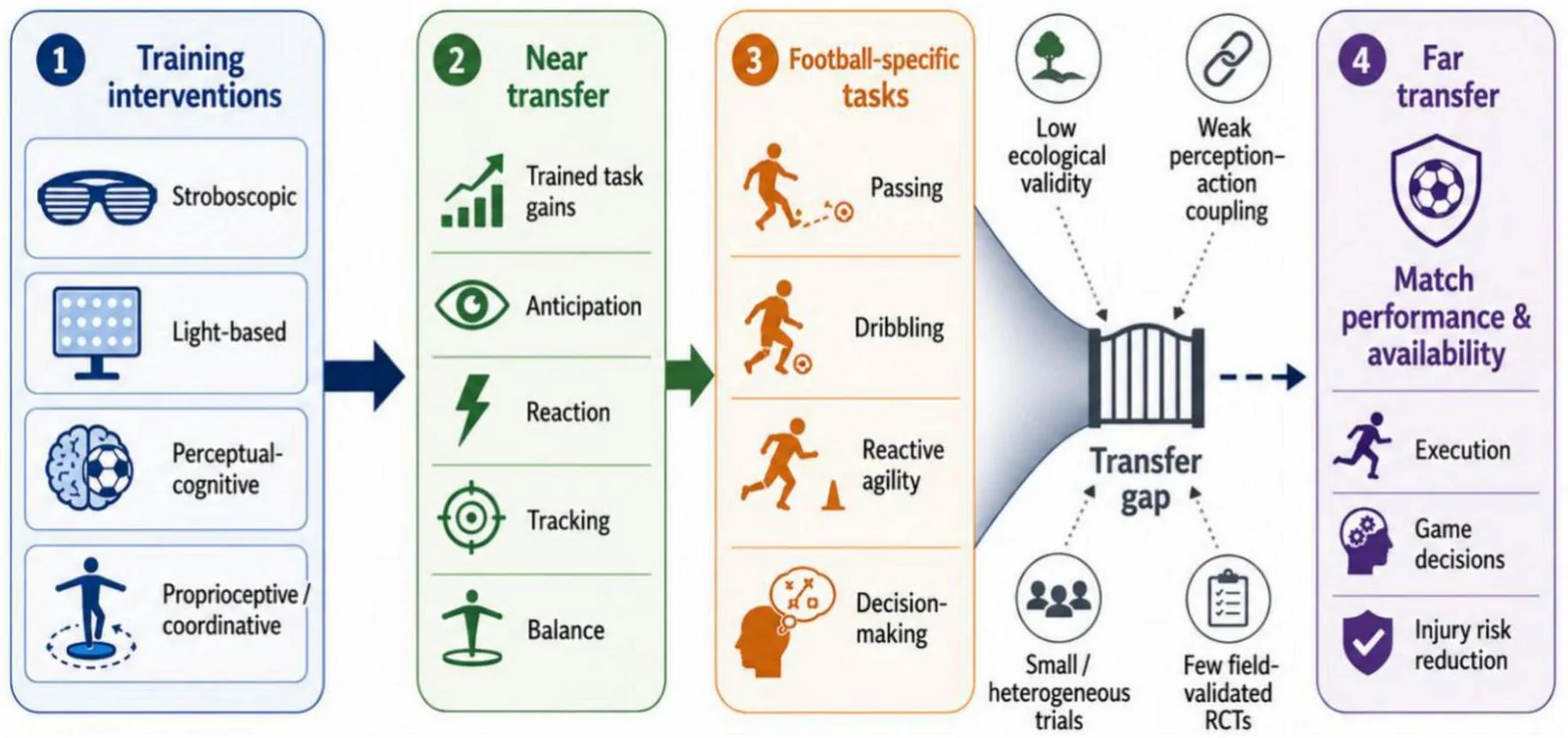
Translational pathway and transfer gap from perceptual-motor training to football-specific performance.

This is the crux of the trainability question. Near-transfer, from training to the trained or a similar laboratory task, is well supported across all three domains. Far-transfer, to competitive performance, is the weak link. The most direct estimate comes from meta-analysis: perceptual-cognitive training improves anticipation and decision-making, but its transfer to real-game performance (g of about 0.65) is markedly smaller than its effect on laboratory performance (g of about 1.51) (Zhu et al., 2024). Task-specific studies point the same way. Light-based reaction speed, though trainable, does not predict field-based reactive agility (Broodryk et al., 2025), and 3D-MOT training reliably raises tracking scores yet does not dependably improve passing decisions (Harenberg et al., 2022). Taken together, these results place the burden of proof on interventions to show effects at the level of the game, not the test. The translational gap between training-induced task improvement and match-level performance transfer is summarized in Figure 3.

The practical implication is a design principle rather than a specific drill. Training should be made representative of match demands, should require sport-specific perceptual and motor responses, and should keep perception coupled to action, with immersive and virtual-reality tools useful insofar as they preserve that coupling (Klatt et al., 2020). The same logic applies to moderators that should be modeled rather than ignored: playing level, position (goalkeeper versus outfield), sex, age and maturation, limb dominance and lateralization, fatigue, and injury status all shape both the abilities and their trainability. These are not nuisance variables to be averaged away: a protocol calibrated to a right-footed senior outfielder may not suit a maturing goalkeeper, and fatigue or injury can invert the sensory weighting a player depends on, as the vision-proprioception findings show (Han et al., 2022). For practitioners, the corollaries are to assess and train the domains together rather than in isolation, to embed perceptual and kinesthetic stimulation inside football-specific tasks, and to monitor availability alongside performance.

The evidence supports one firm conclusion: laboratory improvement is necessary but not sufficient for match improvement, and progress now depends on interventions that keep perception coupled to football-specific action.

## 6 Methodological considerations and limitations

The conclusions above are only as strong as the evidence that supports them, and that evidence has recurring weaknesses that shape how confidently each claim can be held. The problems are of three broad kinds: how the studies are designed, sampled, and measured, what the present review can and cannot establish given how it was conducted, and how the field should be built to answer the questions that matter for practice. The distinction is worth keeping in view, because a finding can be internally sound yet still fail to justify the applied inference a coach or clinician wants to draw from it.

### 6.1 Design, sampling, and measurement constraints

Most performance-related findings are cross-sectional and correlational, samples are frequently small, and the constructs, measures, and terminology are heterogeneous, which makes results difficult to pool across studies. Many assessments rely on low-ecological-validity laboratory tasks whose scores predict match behavior weakly (Van Maarseveen et al., 2018), and the intervention literature contains few randomized controlled trials (RCTs) with on-field performance outcomes, short intervention durations, and a plausible risk of publication bias. The cross-sectional design is the deeper constraint, since it can establish that a capacity accompanies expertise but cannot show that it causes expertise, which is precisely the inference practitioners most want to draw (Van Maarseveen et al., 2018). Even the studies that separate players by level or role do so at a single time point, so an association between, for example, executive function and competitive standing is compatible both with cognition driving selection and with years of training sharpening cognition (Lovecchio et al., 2021; Vestberg et al., 2017). Without longitudinal or experimental leverage, the direction of these relationships stays open, and the applied temptation to select or de-select players on a perceptual-motor score outruns what the data license.

Terminology is a further obstacle, because the same label, such as agility or anticipation, denotes different constructs and different tests across studies, which inflates apparent disagreement and blocks pooling (Broodryk et al., 2025). A choice-reaction test, an instrumented passing task, and a whole-body reactive-agility run all travel under related names yet load on different processes, so nominal agreement across a literature can conceal real divergence in what was measured (Friebe et al., 2024; Hicheur et al., 2020). Sampling compounds the problem. Coverage is uneven: female players, youth-development and non-elite populations, and goalkeeper- and position-specific questions remain under-represented, a gap visible even in otherwise informative work (Katircioglu et al., 2025). Much of the kinesthetic and visuomotor evidence also comes from special populations or single squads, from amputee footballers to one academy cohort, which is useful for internal comparison but limits how far the numbers generalize to the broader playing population (Aytar et al., 2012; Schumacher et al., 2020). Findings drawn largely from right-footed men tested in one country cannot be assumed to hold for women, for left-dominant players, or across the range of maturation and competitive levels at which the sport is played (Neto et al., 2022).

The statistical practices in this area also invite caution. Small samples paired with many candidate visual, cognitive, and motor variables raise the risk of overfitting and of chance associations surviving as reported findings, and preregistration remains uncommon, so the line between confirmatory and exploratory analysis is often blurred (Scharfen and Memmert, 2019; Vestberg et al., 2017). Normative and diagnostic tools inherit the same limits: a battery that separates professionals into performance tiers, or a tracking task that flags concussion, is only as transportable as the sample it was normed on, and thresholds derived from one cohort may not hold in another population or age band (Chermann et al., 2018; Jorge et al., 2026; Lysenko-Martin et al., 2020). Dynamic visual acuity illustrates how measurement conditions themselves move the outcome, since scores depend on target trajectory and contrast rather than on a single fixed value, so protocols that differ in these parameters are not directly comparable (Quevedo-Junyent and Argilés, 2024).

Measurement quality is a specific concern in its own right. The reliability and validity of many sport-vision and proprioception tests are not established, and estimates of transfer differ sharply depending on whether the outcome is a laboratory task or a game measure (Harenberg et al., 2022; Zhu et al., 2024). Proprioceptive measures are especially exposed, because the same joint-position or balance test returns different values before and after exertion or after an acute intervention, so a single score confounds a stable capacity with a transient state (Azevedo et al., 2025; Behan et al., 2018). Without standardized, football-specific, representative batteries reported with test-retest reliability and minimal-detectable-change values, apparent training effects cannot be separated confidently from measurement noise. A related problem is the choice of comparison: without an adequate control for general training, practice, and expectancy, improvement cannot be attributed to the proposed perceptual-motor mechanism, and the size of reported transfer depends heavily on these design choices (Zhu et al., 2024). Several trials in which both the intervention and the comparison group improved illustrate the point, since a gain shared by controls cannot be credited to the specific method under test (Fortes et al., 2025; Trecroci et al., 2022). Effect sizes reported against a trained laboratory task also tend to exceed those reported against a game outcome, so the headline number a study leads with can overstate the benefit a player would see on the pitch (Zhu et al., 2024).

### 6.2 Limitations of this review

Three limitations follow from the approach set out in section “1.1 Scope and approach to the literature” First, the review is narrative. Screening was not duplicated, no protocol was registered, and no risk-of-bias instrument was applied, so selection bias cannot be excluded. We sought and retained findings that cut against the review’s own argument, including the failure of laboratory perceptual-cognitive scores to predict in-situ performance (Van Maarseveen et al., 2018), the absence of a link between light-based reaction speed and field reactive agility (Broodryk et al., 2025), the failure of 3D-MOT training to improve passing decisions (Harenberg et al., 2022), and trials in which control groups improved as much as trained groups (Fortes et al., 2025; Trecroci et al., 2022). Tables 2, 3 nonetheless remain a structured map of the field rather than a complete census.

Second, the imbalance we identify in the primary literature is reproduced in our own reference list. Female footballers are sampled in only a small minority of the studies tabulated here (Katircioglu et al., 2025), and several of the studies carrying most weight are explicitly male-only (Boraczyński et al., 2019; Jorge et al., 2026; Navarro-Santana et al., 2020; Soussi et al., 2025). The conclusions of this review should therefore be read as conclusions about men’s football unless a cited study states otherwise. This is not only a question of representativeness. Sex differences in landing mechanics and anterior cruciate ligament risk are established, and interlimb asymmetry predicts rupture risk in female players specifically (Katircioglu et al., 2025), so kinesthetic screening thresholds and neuromuscular training prescriptions derived from male cohorts cannot be assumed to hold in the women’s game. That part of the sport is professionalizing quickly, and the applied questions this review addresses are if anything more pressing there than in the men’s game. A weaker version of the same caution applies to youth, non-elite, and goalkeeper-specific inference, where the evidence is thinner than the senior elite outfield literature on which most of our conclusions rest.

Third, restricting inclusion to peer-reviewed reports published in English will have excluded relevant work, particularly from national-language sports science literatures. This leaves the synthesis exposed to publication and language bias in the same way as the primary studies it appraises.

### 6.3 Toward field-validated and integrated research

Several directions follow from these constraints. The priority is adequately powered RCTs with match-performance and skill-execution outcomes rather than laboratory endpoints alone, because the recurring failure of the field is not that capacities do not change but that change at the test has not been shown to reach the game (Van Maarseveen et al., 2018; Zhu et al., 2024). Assessment and training should move toward representative and virtual-reality paradigms that preserve perception-action coupling, since soccer-specific virtual environments and immersive peripheral tasks reproduce the spatial and movement demands that generic stimuli strip away (Klatt et al., 2020; Vu et al., 2022). The same logic recommends keeping the trained response coupled to a football action, as the interventions that moved on-field measures did, rather than training a capacity in isolation and expecting it to transfer (Hicheur et al., 2020; Schumacher et al., 2020). Longitudinal talent-development designs are needed to test whether early perceptual-motor profiles forecast later attainment, and trials should report whether gains persist after the intervention ends and whether they appear in match data rather than only in retests of the trained task (Rodrigues et al., 2025).

Integrated multi-domain models should replace single-domain accounts. Because vision, visuomotor control, and kinesthesis operate as one loop, a study that manipulates one stage while ignoring the others risks misattributing effects, as the sensory-reweighting findings between vision and proprioception show (Han et al., 2022; Lee H. et al., 2022). Mechanistic work should proceed alongside outcome trials, because understanding how a program changes perception, coupling, or sensory weighting would explain why some interventions transfer and others do not, and neuroimaging of perceptual-cognitive expertise offers one route into these mechanisms even if it cannot yet guide training directly (DeCouto et al., 2024). Dose is a further under-specified variable: intervention lengths, session frequencies, and total exposures vary widely and are rarely justified, so the field cannot yet say how much of any modality is needed to produce a durable effect (Boraczyński et al., 2019; Navarro-Santana et al., 2020). Protocols should also be individualized to limb dominance, position, and maturation, since the same program need not suit a right-footed senior outfielder and a maturing goalkeeper, and the goalkeeper literature already shows that expertise effects can be context-specific rather than general (Neto et al., 2022; Vouras et al., 2023). Perceptual-motor training should be linked explicitly to injury-availability outcomes, given that the best-evidenced consequence of the kinesthetic domain runs through staying on the pitch, where balance and neuromuscular programs measurably reduce injury risk (Watson et al., 2026; Zheng G et al., 2025). Harmonized reporting standards, including shared construct definitions and full psychometric detail, would let future results be compared and combined rather than accumulating as a set of non-commensurable single studies (Broodryk et al., 2025).

In the interim, three practical recommendations are defensible: treat any single perceptual-motor test with caution for talent identification, use these measures only as part of multidimensional profiling, and train the abilities within a game context rather than in isolation (DeCouto et al., 2024). None of these recommendations requires waiting for perfect evidence, but each should be held provisionally and revised as field-validated data accumulate.

## 7 Conclusion

Visual, visuomotor, and kinesthetic abilities are best understood not as three separate attributes but as coupled stages of one perception-action system that distinguishes football players from non-athletes and relates to how they perform. The strength of that relationship varies by domain and is generally correlational, and it is strongest where basic capacity meets cognitive and motor control rather than in any single measure taken alone. All three domains are trainable at the level of the specific capacities targeted, and this is now well established. The decisive open question is different and narrower: whether trained gains transfer to competitive performance, where the current evidence is weaker for the game than for the test. Near-transfer to trained and laboratory tasks is not in doubt; the gap is far-transfer, and closing it is a question of how training is designed rather than whether these abilities can change at all. Progress depends on assessment and training that are embedded in representative football contexts and on rigorous, field-validated trials that measure what happens on the pitch. For applied work, the safest reading is to use perceptual-motor measures as one input within multidimensional profiling, to train the abilities inside game-representative tasks rather than in isolation, and to track availability alongside performance rather than treating the two as separate concerns. These conclusions rest on an evidence base drawn almost entirely from male players, and their extension to women’s football should be treated as a hypothesis to be tested rather than an assumption. Read this way, the domains are not competing candidates for a single “most important” ability but complementary levers on the same system, with practical value for player development and for the health and availability that underpin sustained performance.
